# Reassessing Antiplatelet Resistance in Ischemic Stroke: Addressing Key Gaps in Precision Medicine Research

**DOI:** 10.1111/cns.70629

**Published:** 2025-10-13

**Authors:** Mufeed Akram Taha

**Affiliations:** ^1^ Department of Medicine, College of Medicine University of Kirkuk Kirkuk Iraq

To Editor in Chief,

1

I recently read the article titled “Prevalence of Aspirin or Clopidogrel Pharmacological Resistance in Ischemic Stroke: A Step Toward Precision Medicine” by Cencer et al., published in *CNS Neuroscience & Therapeutics* (2025) [1], with great interest. This study offers valuable insights into the prevalence of resistance to aspirin and clopidogrel in patients with ischemic stroke and highlights the potential for precision medicine in this area. However, I would like to bring attention to a key weakness that may limit the interpretation and applicability of the findings.

Ischemic stroke is a leading cause of death and disability worldwide, affecting approximately 795,000 individuals annually in the United States alone. Despite advances in acute management, stroke prevention remains a cornerstone of care [2]. Aspirin and clopidogrel are commonly used antiplatelet agents that reduce the risk of recurrent stroke, especially in patients with large vessel atherosclerosis or lacunar strokes [3]. Aspirin exerts its antiplatelet effect by inhibiting cyclooxygenase‐1, thus reducing thromboxane A2 production and platelet aggregation. Clopidogrel, a prodrug activated by the hepatic enzyme *CYP2C19*, inhibits the P2Y12 receptor on platelets, thereby reducing ADP‐mediated platelet activation [4]. These agents, alone or in combination (dual antiplatelet therapy), are widely recommended for secondary stroke prevention, particularly following high‐risk transient ischemic attacks or minor strokes [5].

The study by Cencer et al. contributes to our understanding of the prevalence of aspirin and clopidogrel resistance in stroke patients. However, one significant limitation is the absence of genetic testing for *CYP2C19* polymorphisms. Variations in the *CYP2C19* gene, particularly the *2 and *3 loss‐of‐function alleles, are well‐documented determinants of clopidogrel metabolism and responsiveness. Without routine genotyping, the study cannot distinguish between pharmacodynamic resistance (related to platelet biology) and pharmacokinetic resistance (related to drug metabolism). This omission may have led to an overestimation of clopidogrel resistance in the study population and confounded the observed differences in response by sex and ethnicity.

Additionally, the relatively small sample size and the predominance of Caucasian participants limit the generalizability of the findings, particularly in light of the known variability in *CYP2C19* allele frequencies across different ethnic groups. The single‐center design further raises concerns about selection bias, which may have influenced the study outcomes.

In light of these considerations, future studies should incorporate routine *CYP2C19* genotyping to accurately identify patients at risk of clopidogrel resistance. Large, multicenter studies with diverse populations would also help validate these findings and provide robust evidence to guide individualized antiplatelet therapy for stroke prevention. Such steps would align more closely with the authors' stated goal of advancing precision medicine in the management of ischemic stroke and would enhance the utility of aspirin and clopidogrel in this context.

## Conflicts of Interest

The author declares no conflicts of interest.

## Data Availability

Data sharing not applicable to this article as no datasets were generated or analyzed during the current study.
